# Effects of the Positive Threshold and Data Analysis on Human MOG Antibody Detection by Live Flow Cytometry

**DOI:** 10.3389/fimmu.2020.00119

**Published:** 2020-02-06

**Authors:** Fiona Tea, Deepti Pilli, Sudarshini Ramanathan, Joseph A. Lopez, Vera Merheb, Fiona X. Z. Lee, Alicia Zou, Ganesha Liyanage, Chelsea B. Bassett, Selina Thomsen, Stephen W. Reddel, Michael H. Barnett, David A. Brown, Russell C. Dale, Fabienne Brilot, Australasian New Zealand MOG Study Group

**Affiliations:** ^1^Brain Autoimmunity Group, Kids Neuroscience Centre, Kids Research at the Children's Hospital at Westmead, Sydney, NSW, Australia; ^2^Faculty of Medicine and Health, Discipline of Child and Adolescent Health, The University of Sydney, Sydney, NSW, Australia; ^3^Brain and Mind Centre, The University of Sydney, Sydney, NSW, Australia; ^4^Department of Neurology, Concord Repatriation General Hospital, Sydney, NSW, Australia; ^5^New South Wales Health Pathology, Institute of Clinical Pathology and Medical Research, Westmead Institute for Medical Research, The University of Sydney, Sydney, NSW, Australia; ^6^Faculty of Medicine and Health, School of Medical Sciences, The University of Sydney, Sydney, NSW, Australia

**Keywords:** demyelination, optic neuritis (ON), myelitis, MOG antibody, flow cytometry analysis, antibody detection, patient diagnosis

## Abstract

Human autoantibodies targeting myelin oligodendrocyte glycoprotein (MOG Ab) have become a useful clinical biomarker for the diagnosis of a spectrum of inflammatory demyelinating disorders. Live cell-based assays that detect MOG Ab against conformational MOG are currently the gold standard. Flow cytometry, in which serum binding to MOG-expressing cells and control cells are quantitively evaluated, is a widely used observer-independent, precise, and reliable detection method. However, there is currently no consensus on data analysis; for example, seropositive thresholds have been reported using varying standard deviations above a control cohort. Herein, we used a large cohort of 482 sera including samples from patients with monophasic or relapsing demyelination phenotypes consistent with MOG antibody-associated demyelination and other neurological diseases, as well as healthy controls, and applied a series of published analyses involving a background subtraction (delta) or a division (ratio). Loss of seropositivity and reduced detection sensitivity were observed when MOG ratio analyses or when 10 standard deviation (SD) or an arbitrary number was used to establish the threshold. Background binding and MOG ratio value were negatively correlated, in which patients seronegative by MOG ratio had high non-specific binding, a characteristic of serum that must be acknowledged. Most MOG Ab serostatuses were similar across analyses when optimal thresholds obtained by ROC analyses were used, demonstrating the robust nature and high discriminatory power of flow cytometry cell-based assays. With increased demand to identify MOG Ab-positive patients, a consensus on analysis is vital to improve patient diagnosis and for cross-study comparisons to ultimately define MOG Ab-associated disorders.

## Introduction

Detection of human autoantibodies targeting myelin oligodendrocyte glycoprotein (MOG Ab) is now a relevant and important diagnostic biomarker in the field of central nervous system (CNS) demyelination. MOG Ab-associated disorders encompass a disease entity involving the brain, optic nerve, and spinal cord that is distinct from multiple sclerosis (MS) and aquaporin-4 Ab-positive neuromyelitis optica spectrum disorder (NMOSD) (1–15). The reemergence of MOG Ab in the field of autoimmune diagnostics has sparked wide interest, and with ongoing advances in our understanding of MOG Ab-associated disease, requests for MOG Ab testing have risen dramatically, as treatment regimens and prognosis for MOG Ab-positive patients are divergent from MS and aquaporin-4 Ab-positive NMOSD patients (11, 16, 17). Moreover, some MOG Ab-positive patients, particularly those with relapsing disease or delayed immunotherapy, may accrue residual disability (11, 12, 15–19). As such, early and accurate identification of MOG Ab-seropositivity is crucial.

Detection of human MOG Ab against full-length native conformational MOG using live cell-based assays by flow cytometry or microscopy has been established as the diagnostic gold standard and is superior to assays utilizing fixatives (20–22). Flow cytometry provides an investigator-independent quantitative measure of MOG Ab titers and has been validated and proven reliable, with high sensitivity and specificity (20, 21). Due to the data complexity and non-specific binding in human sera, different analyses of flow cytometry data have been reported. For example, when serum binding to MOG-expressing cells is compared to control cells, quantification of MOG Ab titers has been reported by subtraction (delta) or division (binding ratio). Additionally, there are disparities in calculating the positive threshold. A comparison of published analyses using the same dataset is required to observe whether these variations can influence the assessment of MOG Ab serostatus and patient diagnosis.

Herein, we have used our extensive flow cytometry and clinical published datasets (11, 15, 21) of 482 sera to address the influence of data analysis on the interpretation of MOG Ab serostatus. Furthermore, we make recommendations for the international standardization of flow cytometry-based MOG Ab analysis.

## Materials and Methods

### Patient and Control Samples

In the absence of consensus clinical diagnostic criteria for MOG Ab-associated disorders, sensitivity and specificity were determined from 482 sera divided into two groups: Group A, sera from monophasic and relapsing disorders with reported MOG Ab-association (ADEM, ON, BON, LETM, etc.), and Group B, sera from healthy controls, general medicine, non-inflammatory neurological disorders, demyelinating disorders not associated with MOG Ab (MS, CIS other than ON), and demyelinating disorders not yet associated with MOG Ab (21). Overall, using our own analysis (Analysis 2, Table 1), the dataset included 48 healthy or other neurological disorder patients (24 children and 24 adults, Group B), 47 MOG Ab-negative (MOG Ab-) patients (24 children, 14 in Group A, 10 in Group B, and 23 adults, 8 in Group A, 15 in Group B), 74 adult MS patients (Group B), and 313 MOG Ab-positive (MOG Ab+) sera (151 sera from 123 children, 150 in Group A, 1 in Group B, and 162 sera from 125 adults, 161 in Group A, 1 in Group B). All patient serostatuses have been published, and clinical phenotypes were retrospectively obtained and detailed in (6, 15, 21, 35, 36). The phenotypes of the 25 MOG Ab- patients in Group B (*n* = 10 children, *n* = 15 adults) were included in Supplementary Table 1.

**Table 1:** MOG Ab positivity status across different published flow cytometry analyses.

	**Flow cytometry MOG Ab analysis**				**Pediatric serum**, ***n*** **=** **151^a^** ***n (% total)***		**Adult serum**, ***n*** **=** **162^a^** ***n (% total)***	
	**Quantification of MOG Ab** ***(Seeding of MOG+ and MOG- cells for serum incubation)^b^***	**Positive threshold or cut-off^c^** ***Standard deviations above the mean of controls***	**Controls *n (study)***	**Publications^d^**	**MOG Ab-**	**MOG Ab+**	**MOG Ab-**	**MOG Ab+**
Analysis 1	ΔMOG Mean (Separate wells)	3 SD	24 HC/OND (6) 52 HC/OND (11)	(6, 11)	0 (0)	151 (100)	0 (0)	162 (100)
Analysis 2	ΔMOG Median (Separate wells)	3 SD	28 HC/OND (2) 24 HC/OND (21)	(2, 21)	0 (0)	151 (100)	0 (0)	162 (100)
Analysis 3	ΔMOG Median (Mixed)	(a)3 SD	8 OND	(23, 24)	0 (0)	151 (100)	0 (0)	162 (100)
		(b)6 SD	5 HC	(25)	5 (3)	146 (97)	4 (2)	158 (98)
		(c) 10SD	8 OND	(26, 27)	18 (12)	133 (88)	8 (5)	154 (95)
Analysis 4	Ratio median (Mixed)	>2.5^e^	–	(28, 29)	43 (28)	108 (72)	23 (14)	139 (86)
Analysis 5	Ratio geometric mean (Separate wells)	(a)4 SD	39 HC	(4)	4 (3)	147 (97)	5 (3)	157 (97)
		(b)6 SD	89 OND	(30)	7 (5)	144 (95)	10 (6)	152 (94)
Analysis 6	Ratio Mean (Separate wells)	(a)3 SD	71 OND (3) 23 HC (8)	(3, 8)	10 (7)	141 (93)	25 (15)	137 (85)
		(b)>3^e^	–	(31)	53 (35)	98 (65)	29 (18)	133 (82)
Analysis 7	Ratio Median (Separate wells)	(a)4 SD	14 HC, 19 OND	(32)	14 (9)	137 (91)	20 (12)	142 (88)
		(b)10 SD	30 HC	(33)	64 (42)	87 (58)	57 (35)	105 (65)
Analysis 8	ΔMOG Ratio Mean^f^	>1	–	(34)	17 (11)	134 (89)	7 (4)	155 (96)
Analysis 9	ΔMOG Median (Mixed)	4 SD	24 HC/OND	Recommended	0 (0)	151 (100)	1 (1)	161 (99)
Analysis 10	Ratio Geometric mean (Separate wells)	(a)>2.5	–		40 (26)	111 (74)	25 (15)	137 (85)
		(b)>3	–		66 (44)	85 (56)	34 (21)	128 (79)

a *151 pediatric and 162 adult sera with reported clinical phenotype were included from Tea et al. (21)*.

b *Serum was incubated with MOG-expressing (MOG+) and control (MOG-) cells in independent wells (separate wells) or untransduced MOG+ cells were gated and compared to the MOG+ cells from the same well (mixed)*.

c *Positive threshold calculated using 24 age-matched controls according to published analysis*.

d *Analyses were only included if >10 MOG Ab+ patients were reported and detailed flow cytometry analyses were provided*.

e *Positive threshold determined by an arbitrary number*.

f *ΔMOG/MOG- cells. Seropositivity was reported if a patient is above threshold at least two times in three experiments*.

*HC, Healthy controls; ΔMOG, MOG+ – MOG-; OND, other neurological diseases; Ratio, MOG+/MOG-; SD, standard deviation*.

### Detection of Human MOG Ab by Flow Cytometry

A flow cytometry live cell-based assay was used to detect human serum MOG Ab, as previously described (6, 21, 37). In brief, patient serum (1:50) was incubated with a transduced cell line expressing full-length human MOG, followed by fluorochrome-conjugated anti-human IgG (H+L). Dilution of serum at 1:50 was standard and was most frequently used across studies (2, 3, 6, 8, 23, 24, 30, 32, 38) (Supplementary Table 2). Samples were reported positive if they were above the positive threshold in at least two of three quality-controlled experiments, a feature that may not have been implemented in other studies but ensures a reliable serostatus report and provides insight into serostatus reproducibility (21). MOG-expressing (MOG+) and empty vector control (MOG-) cells incubated with serum in two independent wells were compared in the “separate wells” analysis, and MOG+ cells (~80% transduction rate) were compared to the untransduced cells from the same single well in the “mixed” analysis (Table 1).

### Comparison of Analyses in Determining MOG Ab Positivity

**Figure 1:**
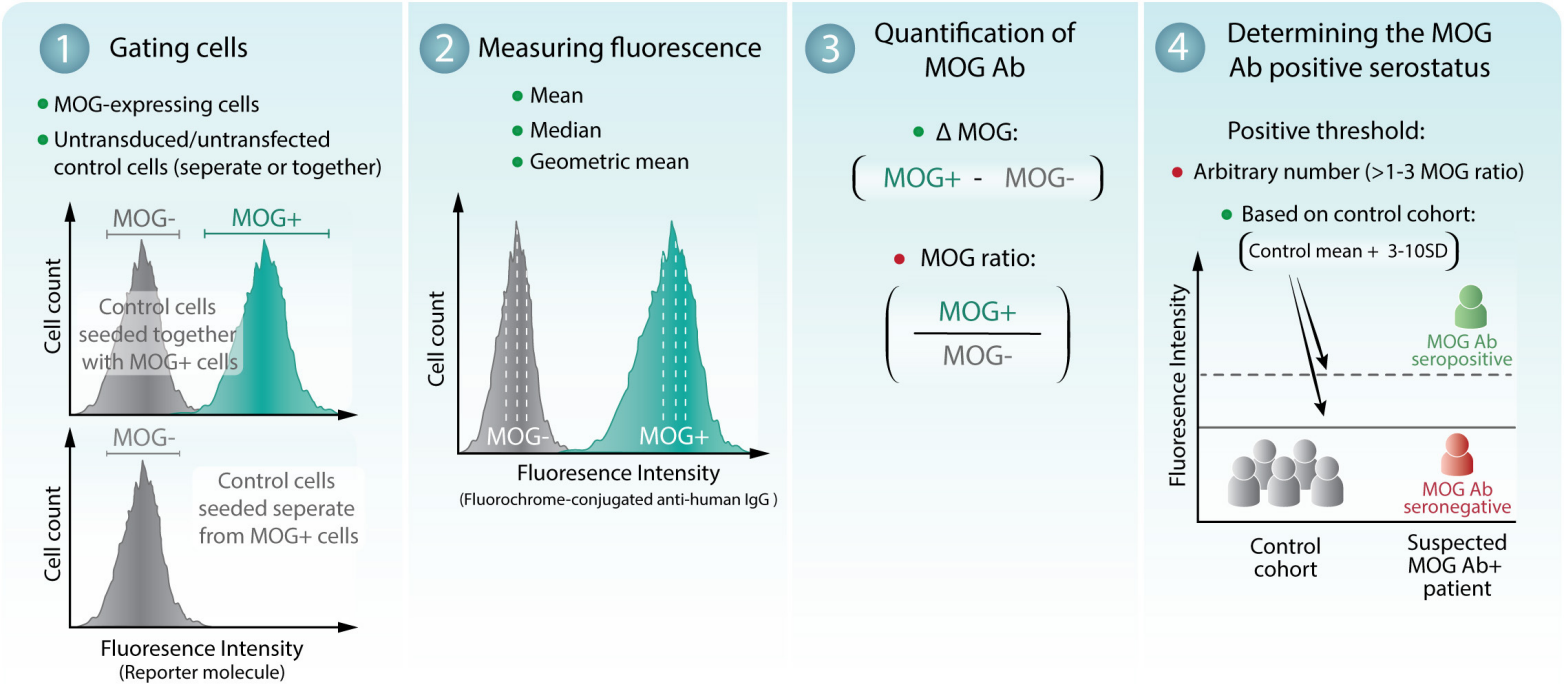
Assessment of patient MOG Ab serostatus by flow cytometry live cell-based assay. (1) MOG-expressing cells (MOG+) and empty vector or untransduced/untransfected control cells (MOG-) were gated. MOG- cells can be either seeded together with or separate from MOG+ cells. (2) The mean, median, or geometric mean fluorescence intensity of the MOG+ and MOG- cells can be determined. (3) MOG Ab binding to MOG is quantified by subtraction (ΔMOG) or division (MOG ratio) of MOG+ and MOG- cells. (4) The threshold of seropositivity can be determined by an arbitrary number or calculated at 3–10 standard deviations (SD) above a control cohort. A breakdown of the analyses is shown in Table 1. Recommended methods of analysis are indicated by green dots. Analyses that demonstrated reduced seropositive outcomes and detection sensitivity are indicated by a red dot.

Assessment of a patient MOG Ab serostatus by flow cytometry can be separated into four stages (Figure 1). (1) Gating of empty vector or untransduced/untransfected MOG- control cells, indicative of serum background binding, and MOG+ cells. Serum can be incubated with MOG- and MOG+ cells seeded together (mixed) or in independent wells (separate). (2) Quantification of sera binding to MOG- and MOG+ cells can be quantified by the median, mean, or geometric mean fluorescence intensity. (3) Determination of MOG Ab binding to MOG by subtraction (ΔMOG, delta); [MOG = Fluorescence of MOG+ cells – Fluorescence of MOG- cells], or division (MOG ratio); $\left[\text{MOG ratio}=\frac{\text{Fluorescence of MOG+ cells}}{\text{Fluorescence of MOG- cells}}\right]$between MOG+ and MOG- cells. (4) Establishing the positive threshold by 3, 4, 6, or 10 standard deviations (SD) above the mean of a control cohort or above an absolute value (Figure 1). Raw flow cytometry datasets were obtained from all patients (*n* = 3 experiments per patient) and reanalyzed using published analyses detailed in Table 1. An age-matched control cohort (*n* = 24), which included patients with general medical and non-inflammatory neurological disorders (and healthy controls in adults), was run concurrently with MOG Ab testing to generate the positive threshold. Published analyses are detailed in Table 1 and were included if the study detailed selection of MOG+ and MOG– cells, quantification of MOG Ab, and threshold calculation and reported at least 10 MOG Ab-positive patients.

In the absence of diagnostic criteria for MOG Ab-associated disorders, sensitivity and specificity analyses were determined using Groups A and B described above (21). Receiver operating characteristic (ROC) curves were generated to evaluate the optimal diagnostic performance of each analysis between these two groups of patients.

### Statistics

Correlation analyses and *R*^2^ values were generated using a linear regression model. Youden's Index, which maximizes sensitivity and specificity, was used to determine the optimal threshold from each ROC curve analysis (39, 40). McNemar's Chi-squared test was used to compare the similarity of the seropositive and seronegative results obtained in the different analyses. McNemar's test compared analyses from the same flow cytometry live dataset. Flow cytometry data were analyzed using FlowJo v10 (TreeStar) software and Microsoft Excel. Figures and schematics were generated using Prism v7.0a (GraphPad Software) and Adobe Illustrator CC 2015 (Adobe Systems).

## Results

### Reduced MOG Ab Seropositivity and Detection Sensitivity by MOG Ratio Analysis

We first compared the serostatus across different published analyses using a flow cytometry live dataset obtained from our previous publications (6, 11, 15, 21) (Table 1). Using a threshold obtained from the control cohort, all patients were determined to be MOG Ab+ when MOG+ and MOG- cells were analyzed from two independent wells (Analyses 1 and 2) or in a single well (Analysis 3a) (151 pediatric and 162 adult samples) (Table 1). There was low intra-assay variability across repeated experiments (Supplementary Table 3), and these analyses led to similar seropositivity results, with high detection sensitivity and specificity (Table 2, Supplementary Table 4). Furthermore, using a ΔMOG and 3SD threshold, MOG Ab positivity serostatus was similar when MOG Ab titers were determined by the mean (Analysis 1), median (Analysis 2), or geometric mean (data not shown) of MOG+ and MOG- cell populations (Tables 1, 2). These results suggest that quantification of MOG Ab binding by flow cytometry is reliable and reproducible and that the practicality of incubating serum with MOG+ and MOG- cells in a single well, rather than two independent wells, could be considered.

**Table 2:** Comparison of sensitivity and specificity of MOG Ab detection across different published flow cytometry analyses.

**Flow cytometry live analysis^a^**		**Children**, ***n*** **=** **199**^****b****^			**Adults**, ***n*** **=** **283**^****c****^		
		**Sensitivity *% (CI)***	**Specificity *% (CI)***	***P*-value^d^**	**Sensitivity *% (CI)***	**Specificity *% (CI)***	***P*-value^d^**
Analysis 1		91.5 (85.8–95.1)	97.1 (83.4–99.9)	1.0	95.3 (90.6–97.8)	95.6 (89.6–98.4)	1.0
Analysis 2		91.5 (85.8–95.1)	97.1 (83.4–99.9)	–	95.3 (90.6–97.8)	95.6 (89.6–98.4)	–
Analysis 3	(a)	91.5 (85.8–95.1)	97.1 (83.4–99.9)	1.0	95.3 (90.6–97.8)	94.7 (87.3–97.3)	1.0
	(b)	88.4 (82.3–92.7)	97.1 (83.4–99.9)	0.073	92.9 (87.6–96.1)	98.2 (93.2–99.7)	1.0
	(c)	80.5 (73.4–86.1)	97.1 (83.4–99.9)	<0.001*	90.5 (84.8–94.3)	99.1 (94.5–100)	0.387
Analysis 4		65.9 (58–73)	100 (87.7–100)	<0.001*	81.7 (74.8–87)	99.1 (94.5–100)	<0.001*
Analysis 5	(a)	89 (83–93.2)	97.1 (83.4–99.9)	0.133	92.3 (86.9–95.7)	98.2 (93.2–99.7)	0.723
	(b)	87.8 (81.6–92.2)	100 (87.7–100)	0.131	89.3 (83.5–93.4)	99.1 (94.5–100)	0.181
Analysis 6	(a)	86 (79.5–90.7)	97.1 (83.4–99.9)	0.03*	80.5 (73.5–86)	97.4 (91.9–99.3)	<0.001*
	(b)	59.8 (51.8–67.2)	100 (87.7–100)	<0.001*	78.1 (71–83.9)	99.1 (94.5–100)	<0.001*
Analysis 7	(a)	83.5 (76.8–88.7)	97.1 (83.4–99.9)	0.003*	83.4 (76.8–88.5)	99.1 (94.5–100)	0.002*
	(b)	53 (45.1–60.8)	100 (87.7–100)	<0.001*	58 (50.2–65.5)	99.1 (94.5–100)	<0.001*
Analysis 8		81.7 (74.8–87.1)	100 (87.7–100)	<0.001*	91.1 (85.5–94.8)	95.6 (89.6–1)	0.023*
Analysis 9		91.5 (85.8–95.1)	97.1 (83.4–99.9)	1.0	94.7 (89.8–97.4)	97.4 (91.9–99.3)	1.0
Analysis 10	(a)	67.7 (59.9–74.6)	100 (83.4–100)	<0.001*	80.5 (73.5–86)	99.1 (94.5–100)	<0.001*
	(b)	51.8 (43.9–59.6)	100 (83.4–100)	<0.001*	75.1 (67.8–81.3)	99.1 (94.5–100)	<0.001*

a *Seropositivity determined by the threshold using 24 age-matched controls according to analyses detailed in Table 1. Cohorts included 164 pediatric^b^ and 169 adult^c^ sera from patients with monophasic and relapsing disorders with reported MOG Ab-association and 35 pediatric^b^ and 114 adult^c^ sera from disorders with no MOG Ab-association yet reported and disorders not associated with MOG Ab (Supplementary Table 3)*.

d P-values determined by McNemar's Chi-squared test with Analysis 2 as the comparator (*P < 0.05). CI = 95% confidence interval.

Among ΔMOG analyses, the seropositivity by a control cohort-based threshold using 3SD (Analysis 1–3a) was not significantly different from 6SD (Analysis 3b) (Table 2). Across analyses that utilized thresholds from 3 to 6 SD above a control cohort-based threshold, MOG ratio analyses (Analysis 5a, 5b, 6a, 7a) showed reduced MOG Ab detection sensitivity (average sensitivity, children 87% ± 2, and adults 87% ± 6) compared to ΔMOG analyses (Analysis 1, 2, 3a, 3b, average sensitivity, children 91% ± 2 and adults 94% ± 1; Table 2, Supplementary Table 4). There was an average seropositive loss of 6% ± 3 in children (average *n* = 10 ± 5, range 5–15) and 11% ± 6 among adults (average *n* = 20 ± 12, range 7–29) across all MOG ratio analyses, which increased with higher SD thresholds (Table 1). When the ratio was determined between ΔMOG and MOG- cells (Analysis 8), detection sensitivity and specificity were high (Table 2); however, the serostatus remained significantly different from that in a ΔMOG analysis (Tables 1, 2). Notably, the MOG ratio determination by geometric mean (Analysis 5) performed the best out of all MOG ratio analyses (Table 1) and performed significantly better than the MOG ratio median with the same 4SD threshold (Analysis 5a vs. 7a, children, *P* = 0.027, adults *P* = 0.001, data not shown; Table 2).

An increasing loss of MOG Ab seropositivity was observed with higher thresholds across all analyses (Table 1). Indeed, when the positive threshold was set 10 SD above the control cohort (Analysis 3c, 7b), there was a ~29% reduced detection sensitivity (Table 2, Supplementary Table 4), and significant loss of seropositivity, which was more pronounced in the MOG ratio (Analysis 7b, *n* = 64 children and *n* = 57 adults reported negative) than ΔMOG analysis (Analysis 3c, *n* = 18 children and *n* = 8 adults reported negative; Table 1). Across all flow analyses, seropositivity loss was the greatest and significantly different from Analysis 2 when an arbitrary threshold was used (MOG ratio > 2.5 or 3, Analysis 4 and 6b), even when a geometric mean was used to quantify MOG Ab binding (Analysis 10; Tables 1, 2). Although it may be hard to compare absolute values directly due to variability in experimental conditions, this suggests that an arbitrary threshold may be difficult to translate across studies.

Overall in children and adults, when using a 3 or 4 SD threshold, the confidence intervals were narrower (Table 2) and sensitivity was higher in the ΔMOG analyses (Analysis 1, 2, and 3a, average sensitivity; 93%) than in the MOG ratio analysis (Analysis 5a, average sensitivity: 91%, 6a: 83%, and 7a: 83%) (Table 2, Supplementary Table 4). Therefore, the ΔMOG value, rather than the MOG ratio, may be a more reliable measure to determine MOG Ab seropositivity. Although sensitivity can be compromised, specificity could be improved by increasing the SDs used to calculate the threshold. Indeed, a ΔMOG median (mixed) analysis using a 4 SD threshold (Analysis 9) showed the highest combined detection sensitivity and specificity (Table 2) and the lowest intra-assay variability (Supplementary Table 3). Furthermore, seropositive results in Analysis 9 were not statistically different from those of Analysis 1 or 2 (Tables 1, 2), but specificity was increased by reducing seropositivity in two MS patients (Supplementary Table 3).

### High Background Binding in Patient Serum Reduces MOG Ab Detection Sensitivity in a Ratio Analysis

**Figure 2:**
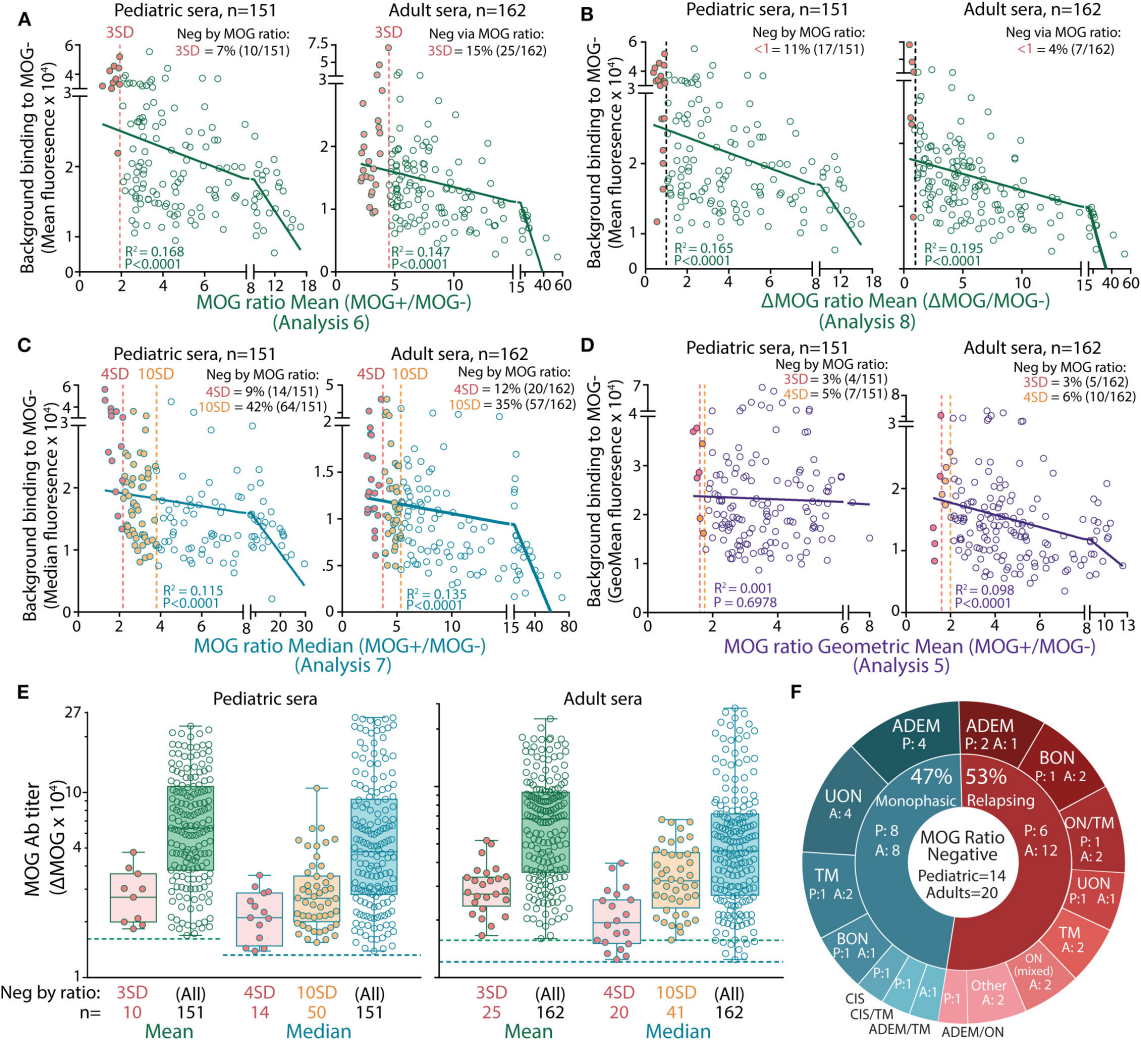
High serum background binding reduced seropositivity detection in MOG ratio analysis. Patients negative (filled red) by mean **(A,B)**, median **(C)**, and geometric mean **(D)** MOG ratio analysis had high background binding. There was a negative correlation between background binding and mean or median MOG ratio values (*P* < 0.0001). **(E)** ΔMOG values of MOG Ab+ patients reported negative in MOG ratio analysis by 3 or 4 SD (red, Analysis 6a and 7a, respectively) or 10 SD (orange, Analysis 7b). Children (left) and adults (right) negative by MOG ratio analysis had a broad range of MOG Ab titers and fell within the range of ΔMOG values of patients who were positive by MOG ratio analysis. Dotted lines represent the ΔMOG positive threshold 3 SD above controls. Representative data from three experiments are shown. **(F)** Patients reported negative by MOG ratio median analysis (4 SD, Analysis 7a) clinically presented with MOG Ab-associated phenotypes. P, pediatric patients; A, adults; ADEM, acute disseminated encephalomyelitis; BON, bilateral optic neuritis; CIS, clinically isolated syndrome; LETM, longitudinally extensive transverse myelitis; ON mixed, combination of BON and UON; ON/TM, simultaneous ON and transverse myelitis; relapsing ADEM, multiphasic ADEM (41); TM, transverse myelitis; UON, unilateral optic neuritis.

Non-specific background serum binding to MOG- cells varied among patients. Analysis by ΔMFI was superior to a ratio analysis, as the MOG ratio was greatly influenced by background binding (Figures 2A,B). Indeed, by MOG ratio analyses, the background binding detected from seronegative samples was significantly higher compared to seropositive samples (children and adults, *P* < 0.0001, Figures 2A,B). There was a negative correlation between background binding and MOG ratio mean, ΔMOG ratio mean, MOG ratio median, and MOG ratio geometric mean, i.e., sera with higher background had lower MOG ratio (*P* < 0.001, Figures 2A–D), further supporting the influence of background binding on reducing detection sensitivity. MOG Ab-positive patients negative by MOG ratio analysis exhibited a wide range of MOG Ab levels when determined by ΔMFI (Figure 2E, red and orange dots), suggesting that MOG Ab titers might not be accurately represented in a MOG ratio in patients with high background binding. Furthermore, among patients of known phenotype, most children and adults negative according to MOG ratio analysis presented with typical MOG Ab-associated phenotypes (Figure 2F). There was no clinical distinction between MOG Ab-positive patients reported to be negative or positive by MOG ratio analyses. Interestingly, although seropositivity results between ΔMOG (Analysis 2) and ratio analyses (Analysis 6–8, 10) were significantly different (*P* < 0.05), Analysis 2 performed similarly to MOG ratio analyses using the geometric mean (Analysis 5, *P* = 0.133 and *P* = 0.723, Table 2).

### Comparisons Against an Optimized Threshold by ROC Analysis

A ROC curve was generated for each analysis, and an optimal threshold with the highest sensitivity and specificity was determined (Supplementary Figure 1, Supplementary Table 5). When the performances of each analysis using the optimal threshold were compared to one another, MOG Ab serostatuses were similar for all analyses with the exception of the MOG ratio Analysis 7 in children and Analysis 6 in adults (data not shown). This demonstrates the high discriminatory power of the flow cytometry dataset when an appropriate positive threshold is used.

## Discussion

Within the expanding field of MOG Ab-associated demyelinating disorders, there are variations in the analysis of flow cytometry data. Precise detection of disease-relevant MOG Ab is essential to advance our understanding of human MOG Ab-associated disorders and implement immunotherapies. Here, we examined the differences across published analyses and demonstrated that high serum background binding in ratio analysis and seropositivity thresholds determined by high SD and arbitrary values reduced detection sensitivity. Furthermore, we showed that the flow cytometry cell-based assay is a robust method with high discriminatory power once appropriate thresholds are utilized.

The human relevance of MOG Ab has been controversial for decades. Fortunately, with a better understanding of the binding characteristics of human MOG Ab, methods of detecting disease-relevant MOG Ab have improved immensely. Microscopy live cell-based assays are widely used (12) but are semi-quantitative and observer-dependent, whereas flow cytometry allows quantification of a broad range of MOG Ab titers, permitting an in-depth comparison between MOG Ab seropositivity analyses. Although other secondary antibodies specific to IgG Fc or IgG1 have been used in the literature, the secondary antibody utilized to generate the flow cytometry dataset in the current study targeted heavy and light IgG chains. However, most of the seropositive patients in our cohort had MOG IgG1 Ab (21), and only a small proportion of patients harbored MOG Ab of the IgM isotype (21, 42). ROC curve analysis and generation of the optimal threshold was used to evaluate the performance of the assay to distinguish disorders with reported MOG Ab association from disorders for which MOG Ab association is not yet reported and disorders not associated with MOG Ab. Flow cytometry analysis demonstrated high specificity and sensitivity among most published analyses with similar seropositive and seronegative reports.

MOG Ab have been demonstrated to be highly sensitive to conformational changes and therefore require the native surface antigen. Once the protein is fixed, in the case of in-house fixatives or commercial kits, assay sensitivity is reduced (20, 21). It is recommended that cells remain live to ensure that conformational MOG epitopes are available for binding. As the assay performance was reliable when MOG-expressing and control cells were incubated together rather than in two independent wells, a pragmatic consideration would be to combine both control and MOG-expressing lines into a single well for acquisition and analysis. Furthermore, although seropositivity between mean, median, and geometric mean was similar, the median or geometric mean value, being more resistant to outliers, represents a truer central value.

We demonstrated reduced MOG Ab seropositivity in a MOG ratio analysis, when the signal from MOG-expressing cells was divided by control cells. This was largely due to the high level of background fluorescence detected on control cells after incubation with some sera. Human serum contains a plethora of proteins and exogenous antigens that could non-specifically bind to cells. As flow cytometry is a highly sensitive method of detection, a broad range of background binding levels can be detected, which will affect the MOG ratio. Although these observations were determined by flow cytometry, these insights can be extended to microscopy, where serum background binding should be critically considered before determining a patient's MOG Ab serostatus.

A common threshold determination across the field is necessary to allow reliable study comparisons. The stringency of the positive cut-off is important in optimizing the sensitivity and specificity of an assay. We showed that an increase in the number of SD values, for example, to 10 SD, changed the performance of the assay, with a notable reduction in sensitivity. Three SDs above a control mean representing the 99th percentile, commonly used in a diagnostic setting, presented with high detection sensitivity and specificity, but 4 SD demonstrated the highest discriminatory power in a ΔMOG median analysis. Although there is a broad range of MOG Ab titers, the data are not normally distributed (21) and are “bottom-heavy;” therefore, the serostatus of patients with MOG Ab titers close to threshold are more susceptible to threshold changes. If a control cohort was tested alongside the patient samples, which was the case for many flow cytometry analyses, a ΔMOG analysis is recommended. As arbitrary thresholds may not be accurately translated across studies, an independent threshold should be generated for each experiment to account for inter-assay variability. However, a ratio analysis can be advantageous when a control cohort is not available. The geometric mean normalizes skewed data and is most appropriate in the quantification of ratios. We showed that the geometric mean MOG ratio analysis was similar to the ΔMOG median analysis and demonstrated that ratio values could discriminate disease from non-disease when an optimal threshold by ROC analysis was used. Once this threshold is validated in several cohorts, the geometric mean MOG ratio could be an alternative if a control cohort is not available.

A limitation of this study was that the threshold for all analyses was generated with 24 controls, although the number of control samples used to establish the threshold in the published analyses varied. However, the 24 controls in the current study generated a stringent threshold for all analyses. Furthermore, the most frequent dilution across studies was similar to the one used to generate our dataset, but other studies have tested a range of different dilutions. Although assessing the effect of different serum dilutions was outside the scope of this study, the influence of high serum background in flow cytometry remains useful.

As MOG Ab are becoming a prevalent diagnostic biomarker, these results highlight caveats in using a binding ratio and prompt an international agreement on data analysis, which will permit direct comparisons between studies and streamline diagnosis of MOG Ab-associated disease.

### Recommendations for MOG Ab Analysis by Flow Cytometry

1. MOG-expressing cells can be incubated in the same well as control cells. Fluorescence intensity of control or MOG-expressing cells can be calculated by the mean, median, or geometric mean.
2. A positive threshold determined by a control threshold generated in each experimental run is ideal. MOG Ab binding to MOG should be calculated using ΔMOG instead of a MOG ratio if a control cohort is available.
3. Ratio analysis using the geometric mean could be utilized if a control cohort is unavailable. The optimal threshold by ROC curve analysis should be validated before implementation.
4. Additional parameters may vary, such as serum dilution, secondary antibody, and flow cytometry experimental conditions. Although these recommendations are based on a serum dilution of 1:50 and detection of IgG (H+L), these concepts can be applicable to all flow cytometry analyses.

## Data Availability Statement

The datasets presented in the current study are available from the corresponding author upon reasonable request.

## Ethics Statement

The studies involving human participants were reviewed and approved by Sydney Children's Hospitals Network (NEAF 12/SCHN/395). Informed consent to participate in this study was provided by the participants or their legal guardian/next of kin.

## Author Contributions

FT and FB designed the study. The Australasian and New Zealand MOG Study Group enrolled and managed patients. FT, SR, and DP conducted experiments and analyses. FT wrote the first draft of the manuscript and prepared figures and tables. FB designed and coordinated research and verified results. All authors reviewed the draft before submission.

### Conflict of Interest

The authors declare that the research was conducted in the absence of any commercial or financial relationships that could be construed as a potential conflict of interest.
