# Supplementary material for: Effects of the Positive Threshold and Data Analysis on Human MOG Antibody Detection by Live Flow Cytometry
Source: Front Immunol. 2020 Feb 6;11:119. doi: 10.3389/fimmu.2020.00119 (PMC7016080; doi:10.3389/fimmu.2020.00119)
Supplement: Supplementary file 1 [file Data_Sheet_1.docx]

# Supplementary Data

**Supplementary Table 1.** Patient phenotypes of MOG Ab-negative from Group B. IQR = interquartile range, CIS = clinically isolated syndrome, ON = optic neuritis, MOG Ab- = MOG antibody negative, MOG Ab+ = MOG antibody positive, MS = multiple sclerosis.

| Child or Adult | *N (% Total)* | Sex  *Female (% Female)* | Age  *Median (IQR)* | Phenotype |
| --- | --- | --- | --- | --- |
| Child, N=10 | 5 (50) | 2 (40) | 15 (3-17) | CIS non-ON |
|  | 2 (20) | 0 (0) | 15 (-) | Encephalitis |
|  | 2 (20) | 1 (50) | 16 (-) | MS |
|  | 1 (10) | 1 (100) | 8 (-) | Brainstem glioma |
| Adults, N=15 | 10 (67) | 8 (80) | 39 (25-49) | MS |
|  | 1 (7) | 0 (0) | 78 (-) | Infectious prodrome |
|  | 1 (7) | 1 (100) | 51 (-) | Migraine |
|  | 1 (7) | 1 (100) | 48 (-) | Neuropathy |
|  | 1 (7) | 0 (0) | 32 (-) | Rhombencephalitis |
|  | 1 (7) | 1 (100) | 47 (-) | White matter lesion |

**Supplementary Table 2.** Variability of flow cytometry parameters across different MOG Ab detection studies. ^a^Breakdown of analyses is shown in Table 1. ^b^Publications were only included if more than 10 MOG Ab+ patients were reported and detailed flow cytometry analyses were provided. ^c^Isotype of the secondary antibody anti-IgG not specified. AF647 = Alexa Fluor 647, APC = allophycocyanin, BD = Becton Dickinson, hMOG = human MOG, NS = not specified, PE = phycoerythrin.

| Flow cytometry analysis^a^ | | Publication^b^ | Serum Dilution | Secondary fluorochrome-conjugated detection antibody | | Flow cytometer | Analysis Software |
| --- | --- | --- | --- | --- | --- | --- | --- |
| Analysis 1 |  | ([6](#_ENREF_6)) | 31 | 1:50 | AF647-conjugated goat anti-human IgG H+L (Invitrogen) | BD LSR-II | FlowJo® v7.5 |
|  |  | ([11](#_ENREF_11)) | 59 | 1:50 | AF647-conjugated goat anti-human IgG H+L (Invitrogen) | BD LSR-II | FlowJo® v7.5 |
| Analysis 2 |  | ([21](#_ENREF_21)) | 287 | 1:50 | AF647-conjugated goat anti-human IgG (H+L) (Invitrogen) | BD LSR-II | FlowJo® v10 |
|  |  | ([2](#_ENREF_2)) | 22 | 1:100 | AF488‐conjugated goat anti‐human IgG (H+L) (Invitrogen) | BD LSR-II | FlowJo® v7.5 |
| Analysis 3 | (a) | ([26](#_ENREF_26)) | 31 ([26](#_ENREF_26))  21 ([27](#_ENREF_27)) | 1:50 | APC-conjugated goat anti-human IgG^c^ (Jackson ImmunoResearch) | BD LSR-II | BD FACSDiva |
|  | (b) | ([35](#_ENREF_35)) | 15 | 1:10 | AF488 goat anti-human IgG (H+L) (Invitrogen) | BD Calibur | - |
|  | (c) | ([36](#_ENREF_36)) | 20 | 1:200 | APC-conjugated goat anti-human IgG^c^ (Jackson ImmunoResearch) | BD LSR-II | BD FACSDiva |
|  |  | ([37](#_ENREF_37)) | 31 | 1:50 | APC-conjugated goat anti-human IgG^c^ (Jackson ImmunoResearch) | BD LSR-II | BD FACSDiva |
| Analysis 4 |  | ([38](#_ENREF_38)) | 27 | NS | AF647−conjugated anti-human IgG1 Fc region-specific (Southern Biotech) | NS | NS |
|  |  | ([39](#_ENREF_39)) | 25 | 1:20 | AF647–conjugated mouse anti-human IgG1 Fc region–specific (Southern Biotech) | NS | NS |
| Analysis 5 | (a) | ([4](#_ENREF_4)) | 31 | 1:50 | PE-conjugated anti-human IgG^c^ (Jackson ImmunoResearch) | NS | NS |
|  | (b) | ([28](#_ENREF_28)) | 11 | 1:50 | FITC-conjugated mouse anti-human IgG1 (SouthernBiotech) | NS | NS |
| Analysis 6 | (a) | ([8](#_ENREF_8)) | 13 | 1:50 | Biotin-conjugated anti-human IgG^c^ followed by streptavidin-PE (Invitrogen) | BD LSR-II | Flowjo® v8.8.6 |
|  |  | ([3](#_ENREF_3)) | 39 | 1:50 | PE-conjugated F (ab’) 2-fragment donkey anti-human IgG (Jackson ImmunoResearch) | BD LSR-II | Flowjo® |
|  | (b) | ([40](#_ENREF_40)) | 24 | 1:128 | PerCP-Cy5.5-conjugated goat anti-human IgG^c^ (Biolegend) | BD Calibur | NS |
| Analysis 7 | (a) | ([29](#_ENREF_29)) | 111 | 1:50 | Biotin-SP-conjugated goat anti-human IgG^c^ followed by streptavidin-Dy light 649 (Jackson ImmunoResearch) | NS | NS |
|  | (b) | ([41](#_ENREF_41)) | 24 | 1:5 | APC-conjugated anti-human-IgG1 (Miltenyi Biotec) | BD Canto II | FlowJo® v10.4.1 |
| Analysis 8 |  | ([42](#_ENREF_42)) | 79 | 1:320 | APC-conjugated goat anti human IgG-Fcγ fragment specific (Jackson ImmunoResearch) | NS | NS |

**Supplementary Table 3.** Variability of MOG Ab positivity status across different flow cytometry experiments.

^a^151 pediatric and 162 adult MOG Ab seropositive samples with reported clinical phenotype were included from Tea et al., 2019. Breakdown of analyses is shown in Table 1.

|  |  | Pediatric serum, n=151^a^ | | | | | | Adult serum, n=162^a^ | | | | | | | | |
| --- | --- | --- | --- | --- | --- | --- | --- | --- | --- | --- | --- | --- | --- | --- | --- | --- |
|  |  | Times MOG Ab+ from 3 experiments  *n samples* | | | | MOG Ab serostatus  *N (% total)* | | Times MOG Ab+ from 3 experiments  *n samples* | | | | | | | MOG Ab serostatus  *N (% total)* | |
|  |  | 0 | 1+ | 2+ | 3+ | MOG Ab- | MOG Ab+ | 0 | | 1+ | | 2+ | | 3+ | MOG Ab- | MOG Ab+ |
| Analysis 1 |  | 0 | 0 | 7 | 144 | 0 (0) | 151 (100) | 0 | 0 | | 6 | | 156 | | 0 (0) | 162 (100) |
| Analysis 2 |  | 0 | 0 | 8 | 143 | 0 (0) | 151 (100) | 0 | 0 | | 6 | | 156 | | 0 (0) | 162 (100) |
| Analysis 3 | (a) | 0 | 0 | 1 | 150 | 0 (0) | 151 (100) | 0 | 0 | | 2 | | 160 | | 0 (0) | 162 (100) |
|  | (b) | 2 | 3 | 5 | 141 | 5 (3) | 146 (97) | 1 | 3 | | 3 | | 155 | | 4 (2) | 158 (98) |
|  | (c) | 11 | 7 | 11 | 122 | 18 (12) | 133 (88) | 3 | 5 | | 7 | | 147 | | 8 (5) | 154 (95) |
| Analysis 4 |  | 24 | 19 | 10 | 98 | 43 (28) | 108 (72) | 10 | 13 | | 29 | | 110 | | 23 (14) | 139 (86) |
| Analysis 5 | (a) | 0 | 4 | 2 | 145 | 4 (3) | 147 (97) | 1 | 4 | | 5 | | 152 | | 5 (3) | 157 (97) |
|  | (b) | 2 | 5 | 4 | 140 | 7 (5) | 144 (95) | 5 | 5 | | 11 | | 141 | | 10 (6) | 152 (94) |
| Analysis 6 | (a) | 1 | 9 | 7 | 134 | 10 (7) | 141 (93) | 5 | 20 | | 22 | | 115 | | 25 (15) | 137 (85) |
|  | (b) | 26 | 27 | 22 | 76 | 53 (35) | 98 (65) | 17 | 12 | | 17 | | 116 | | 29 (18) | 133 (82) |
| Analysis 7 | (a) | 5 | 9 | 15 | 122 | 14 (9) | 137 (91) | 9 | 11 | | 19 | | 123 | | 20 (12) | 142 (88) |
|  | (b) | 30 | 34 | 12 | 75 | 64 (42) | 87 (58) | 25 | 32 | | 28 | | 77 | | 57 (35) | 105 (65) |
| Analysis 8 | | 5 | 12 | 15 | 119 | 17 (11) | 134 (89) | 4 | 3 | | 8 | | 147 | | 7 (4) | 155 (96) |
| Analysis 9 | | 0 | 0 | 3 | 148 | 0 (0) | 151 (100) | 1 | 0 | | 4 | | 157 | | 1 (1) | 161 (99) |
| Analysis 10 | (a) | 18 | 22 | 15 | 96 | 40 (26) | 111 (74) | 12 | 13 | | 17 | | 120 | | 25 (15) | 137 (85) |
|  | (b) | 39 | 27 | 14 | 71 | 66 (44) | 85 (56) | 19 | 15 | | 34 | | 94 | | 34 (21) | 128 (79) |

**Supplementary Table 4.** MOG Ab seropositivity across all patient groups.

Serostatus was determined from three experiments and comparison between patients with monophasic and relapsing disorders with reported MOG Ab-association, and disorders with not yet reported MOG Ab-association and disorders not associated with MOG Ab. Groups are described in Materials and Methods.

|  |  | Children  *n seropositive (% of total)* | | | | | Adults  *n seropositive (% of total)* | | | | | | |
| --- | --- | --- | --- | --- | --- | --- | --- | --- | --- | --- | --- | --- | --- |
|  |  | Controls, n=24 | MOG Ab-, n=24 | MOG Ab+, n=151 | MOG Ab associated phenotype, n=164 | Non-MOG Ab associated phenotype, n=35 | | Controls, n=24 | MOG Ab-, n=23 | MS, n=74 | MOG Ab+, n=162 | MOG Ab associated phenotype, n=169 | Non-MOG Ab associated phenotype, n=114 |
| Analysis 1 |  | 0 (0) | 0 (0) | 151 (100) | 150 (91) | 1 (3) | | 0 (0) | 0 (0) | 4 (5) | 162 (100) | 161 (95) | 5 (4) |
| Analysis 2 |  | 0 (0) | 0 (0) | 151 (100) | 150 (91) | 1 (3) | | 0 (0) | 0 (0) | 4 (5) | 162 (100) | 161 (95) | 5 (4) |
| Analysis 3 | (a) | 0 (0) | 0 (0) | 151 (100) | 150 (91) | 1 (3) | | 0 (0) | 0 (0) | 5 (7) | 162 (100) | 161 (95) | 6 (5) |
|  | (b) | 0 (0) | 0 (0) | 146 (97) | 145 (88) | 1 (3) | | 0 (0) | 0 (0) | 1 (1) | 158 (98) | 157 (93) | 2 (2) |
|  | (c) | 0 (0) | 0 (0) | 133 (88) | 132 (80) | 1 (3) | | 0 (0) | 0 (0) | 0 (0) | 154 (95) | 153 (91) | 1 (1) |
| Analysis 4 |  | 0 (0) | 0 (0) | 108 (72) | 108 (66) | 0 (0) | | 0 (0) | 0 (0) | 0 (0) | 139 (86) | 138 (82) | 1 (1) |
| Analysis 5 | (a) | 0 (0) | 0 (0) | 146 (97) | 146 (89) | 1 (3) | | 0 (0) | 0 (0) | 1 (1) | 157 (97) | 156 (92) | 2 (2) |
|  | (b) | 0 (0) | 0 (0) | 144 (95) | 144 (188) | 0 (0) | | 0 (0) | 0 (0) | 0 (0) | 152 (94) | 151 (89) | 1 (1) |
| Analysis 6 | (a) | 0 (0) | 0 (0) | 141 (93) | 141 (86) | 0 (0) | | 0 (0) | 1 (1) | 1 (1) | 137 (85) | 136 (80) | 3 (3) |
|  | (b) | 0 (0) | 0 (0) | 98 (65) | 98 (60) | 0 (0) | | 0 (0) | 0 (0) | 0 (0) | 133 (82) | 132 (78) | 1 (1) |
| Analysis 7 | (a) | 0 (0) | 0 (0) | 137 (91) | 137 (84) | 0 (0) | | 0 (0) | 0 (0) | 0 (0) | 142 (88) | 141 (83) | 1 (1) |
|  | (b) | 0 (0) | 0 (0) | 87 (58) | 87 (53) | 0 (0) | | 0 (0) | 0 (0) | 0 (0) | 105 (65) | 104 (62) | 1 (1) |
| Analysis 8 | | 0 (0) | 0 (0) | 134 (89) | 134 (82) | 0 (0) | | 0 (0) | 0 (0) | 4 (5) | 155 (96) | 154 (91) | 5 (4) |
| Analysis 9 | | 0 (0) | 0 (0) | 150 (99) | 149 (91) | 1 (3) | | 0 (0) | 0 (0) | 0 (0) | 161 (99) | 160 (95) | 3 (3) |
| Analysis 10 | (a) | 0 (0) | 0 (0) | 111 (74) | 111 (100) | 0 (0) | | 0 (0) | 0 (0) | 0 (0) | 137 (85) | 136 (80) | 1 (1) |
|  | (b) | 0 (0) | 0 (0) | 85 (56) | 85 (100) | 0 (0) | | 0 (0) | 0 (0) | 0 (0) | 128 (79) | 127 (75) | 1 (1) |

**Supplementary Table 5.** Comparison of published analyses after determination of the optimal threshold by ROC analysis.

Sensitivity and specificity were determined by comparison of patients with monophasic and relapsing disorders with reported MOG Ab-association, and disorders with not yet reported MOG Ab-association and disorders not associated with MOG Ab (Supplementary Table 2). ^a^The optimal positive threshold was determined by the highest combined specificity and sensitivity. Breakdown of analyses is included in Table 1.

|  | Children | | | | | Adults | | | | |
| --- | --- | --- | --- | --- | --- | --- | --- | --- | --- | --- |
|  | Optimal  threshold^a^ | Sensitivity  % | Specificity  % | MOG Ab associated phenotype, n=164 | Non-MOG Ab associated phenotype, n=35 | Optimal  threshold^a^ | Sensitivity  % | Specificity  % | MOG Ab associated phenotype, n=169 | Non-MOG Ab associated phenotype, n=114 |
| Analysis 1 | > 13439 | 92.68 | 97.14 | 152 (93) | 1 (3) | > 15883 | 95.27 | 99.12 | 161 (95) | 1 (1) |
| Analysis 2 | > 11326 | 92.07 | 97.14 | 150 (91) | 1 (3) | > 12312 | 95.27 | 96.49 | 161 (95) | 4 (4) |
| Analysis 3a, 3b, 3c, 8 | > 7859 | 92.07 | 97.14 | 151 (92) | 1 (3) | > 9455 | 94.67 | 98.25 | 160 (95) | 2(2) |
| Analysis 4 | > 1.479 | 91.88 | 97.14 | 151 (92) | 1 (3) | > 1.727 | 93.45 | 97.35 | 158 (93) | 4 (4) |
| Analysis 5a, 5b, 9a, 9b | > 1.6 | 91.88 | 100 | 149 (91) | 0 (0) | > 1.751 | 94.05 | 96.46 | 156 (92) | 4 (4) |
| Analysis 6a, 6b | > 1.725 | 89.63 | 97.14 | 147 (90) | 1 (3) | > 2.135 | 95.27 | 91.23 | 161 (95) | 10 (9) |
| Analysis 7a, 7b | > 1.9 | 86.59 | 100 | 142 (87) | 0 (0) | > 2.15 | 95.27 | 96.49 | 161 (95) | 4 (4) |
| Analysis 8 | > 0.5925 | 90.85 | 94.29 | 149 (91) | 2 (6) | > 1.059 | 93.36 | 95.61 | 154 (91) | 5 (4) |

**
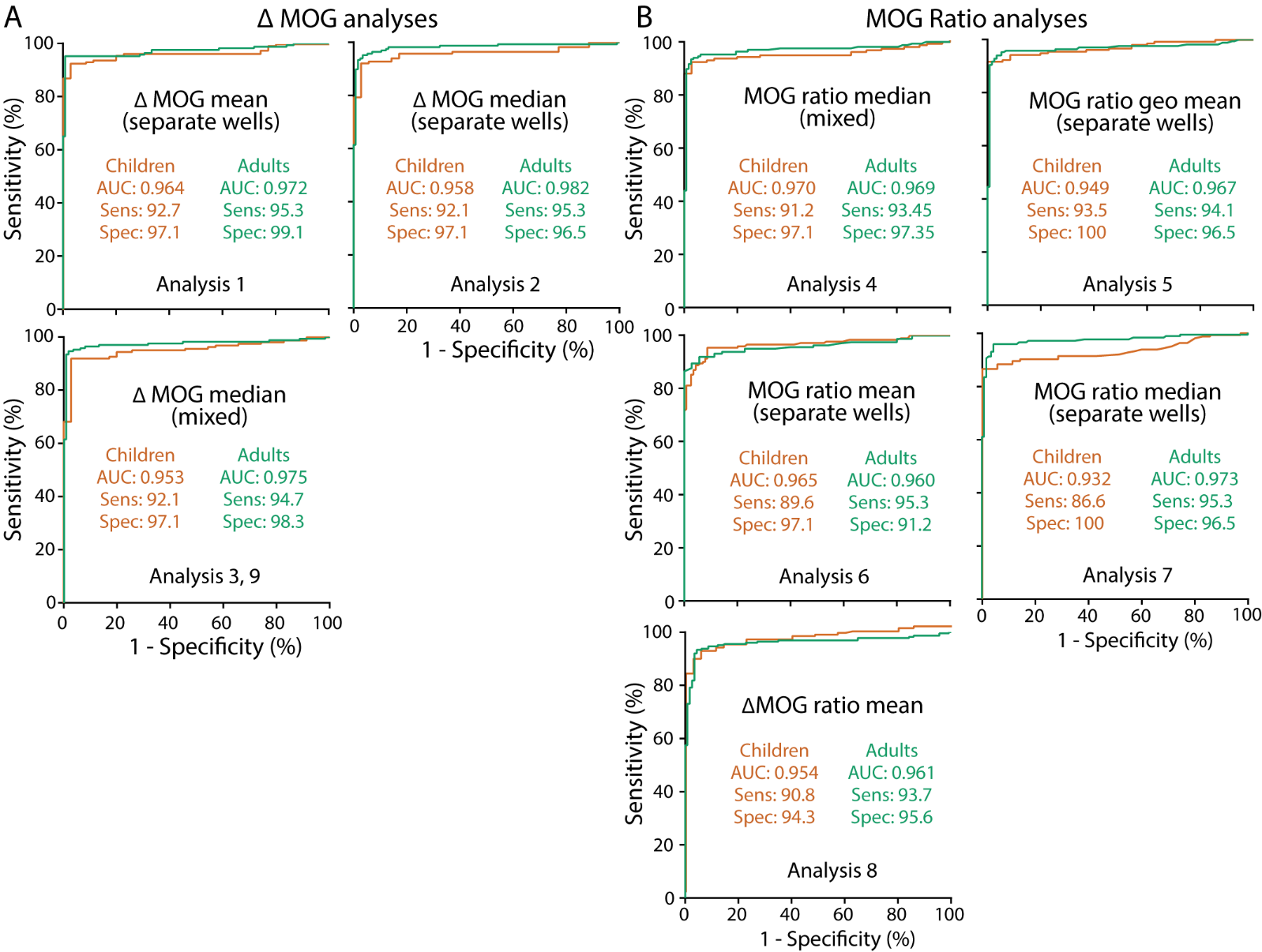
**

**Supplementary Figure 1.** Receiver operating curve (ROC) analysis of published flow cytometry analyses. ROC analyses were generated for (**A**) Δ MOG and (**B**) ratio analyses by comparing patients with disorders with reported MOG Ab-association (164 pediatric and 169 adult sera) from disorders with not yet reported MOG Ab-association and disorders not associated with MOG Ab (35 pediatric and 114 adult sera). An optimal threshold was obtained by maximizing detection sensitivity and specificity. The area under the curve (AUC), sensitivity, and specificity from the optimal threshold were high and similar across all analyses except in children ratio Analysis 7 and adults ratio Analysis 6 (McNemar’s Test, data not shown). Breakdown of analyses is included in Table 1.
