# Supplementary material for: Effects of the Positive Threshold and Data Analysis on Human MOG Antibody Detection by Live Flow Cytometry
Source: Front Immunol. 2020 Feb 6;11:119. doi: 10.3389/fimmu.2020.00119 (PMC7016080; doi:10.3389/fimmu.2020.00119)
Supplement: Supplementary file 2 [file Data_Sheet_2.docx]

# Appendix: Study group author list: the Australasian and New Zealand MOG Study Group

Adriane, Sinclair, Neurosciences Unit, Queensland Children’s Hospital, University of Queensland, Brisbane, Australia.

Allan, G., Kermode, Perron Institute, University of Western Australia, Perth, Australia.

Andrew, P. D., Henderson, Department of Neurology, Westmead Hospital, Sydney, Australia.

Andrew, Kornberg, Department of Neurology, Royal Children’s Hospital, Melbourne, Australia.

Anneke, van der Walt, Department of Neurosciences, Central Clinical School, Monash University, Melbourne, Australia.

Annie, Bye, Department of Neurology, Sydney Children’s Hospital, Sydney, Australia

Anthony, Fok, Department of Neurology, Monash Health, Melbourne, Australia.

Benjamin, McGettigan, Department of Immunology, Fiona Stanley Hospital, Perth, Australia.

Benjamin, Trewin, Brain and Mind Centre, University of Sydney, Sydney, Australia.

Bruce, Brew, Department of Neurology, St Vincent’s Hospital, Sydney, Australia.

Bruce, Taylor, Department of Neurology, Royal Hobart Hospital, Hobart, Australia.

Chris, Bundell, Department of Immunology, PathWest, Perth, Australia.

Christina, Miteff, Department of Neurology, John Hunter Children’s Hospital, Newcastle, Australia.

Christopher, Troedson, TY Nelson Department of Neurology and Neurosurgery, Children’s Hospital at Westmead, Sydney, Australia.

Clair, Pridmore, Women and Children’s Hospital, Adelaide, Australia.

Claire, Spooner, Paediatric Neuroservices, Starship Children’s Health, Auckland District Health Board, Auckland, New Zealand.

Clare, L., Fraser, Save Sight Institute, Faculty of Medicine and Health, The University of Sydney, Sydney, Australia.

Con, Yiannikas, Faculty of Medicine and Health, The University of Sydney, Sydney, Australia.

Cullen, O'Gorman, Princess Alexandra Hospital and School of Medicine, University of Queensland, Brisbane, Australia.

Damian, Clark, Women and Children’s Hospital, Adelaide, Australia.

Dan, Suan, Westmead Clinical School, University of Sydney and Garvan Institute of Medical Research, Sydney, Australia.

David, Booth, Westmead Institute for Medical Research, Sydney, Australia

Dean, Jones, School of Medicine, University of Tasmania and Department of Neurology, Royal Hobart Hospital, Hobart, Australia.

Dean, Kilfoyle, Department of Neurology, Auckland Hospital, Auckland, New Zealand.

Deepak, Gill, TY Nelson Department of Neurology and Neurosurgery, Children’s Hospital at Westmead, Sydney, Australia.

Denis, Wakefield, Department of Immunology, St George Hospital and the University of New South Wales Sydney, Australia.

Dirk, Hofmann, Flinders Medical Centre and Flinders University, Adelaide, Australia.

Emily, Mathey, Brain and Mind Centre, University of Sydney, Sydney, Australia.

Eppie, M. Yiu, Department of Neurology, Royal Children’s Hospital and Neurosciences Research, Murdoch Children’s Research Institute and Department of Pediatrics, The University of Melbourne.

Ernest, Willoughby, Department of Neurology, Auckland City Hospital, Grafton, New Zealand

Esther, M. Tantsis, Brain Autoimmunity Group, Kids Neuroscience Centre, Kids Research at the Children’s Hospital at Westmead, Sydney, Australia.

Fiona, McKay, Westmead Institute for Medical Research, Sydney, Australia

Gina, O'Grady, Paediatric Neuroservices, Starship Children’s Health, Auckland District Health Board, Auckland, New Zealand.

Grant, Parnell, Westmead Institute for Medical Research, Sydney, Australia

Hannah, F., Jones, TY Nelson Department of Neurology and Neurosurgery, Children’s Hospital at Westmead, Sydney, Australia.

Heidi, Beadnall, Brain and Mind Centre, University of Sydney, Sydney, Australia.

Helmut, Butzkueven, Department of Neurosciences, Monash University, Melbourne, Australia.

Ian, Andrews, Department of Neurology, Sydney Children’s Hospital, Sydney, Australia.

Ian, Sutton, Department of Neurology, St Vincent’s Hospital, Sydney, Australia.

Jeanette, Lechner-Scott, Hunter Medical Research Institute, Faculty of Medicine and Public Health, The University of Newcastle.

Jennifer, MacIntyre, Department of Neurology, Royal Hobart Hospital, Hobart, Australia.

Jennifer, M., Sandbach, Department of Ophthalmology, Prince of Wales Hospital, Sydney, Australia.

Jeremy, Freeman, Department of Neurology, Royal Children’s Hospital, Melbourne, Australia.

John, King, Department of Neurology, Royal Melbourne Hospital, Melbourne, Australia.

John, H., O'Neill, Department of Neurology, St Vincent’s Hospital, Sydney, Australia.

John, Parratt, Department of Neurology, Royal North Shore Hospital, Sydney, Australia.

Joshua, Barton, Brain and Mind Centre, University of Sydney, Sydney, Australia.

Justin, Garber, Brain and Mind Centre, University of Sydney, Sydney, Australia.

Kate, Ahmad, Department of Neurology, Royal North Shore Hospital, Sydney, Australia.

Kate, Riney, Neurosciences Unit, Queensland Children’s Hospital/University of Queensland, Brisbane, Australia.

Katherine, Buzzard, Eastern Health Clinical School, Monash University, Box Hill Hospital, Melbourne, Australia.

Kavitha, Kothur, TY Nelson Department of Neurology and Neurosurgery, Children’s Hospital at Westmead, Sydney, Australia.

Mahtab, Ghadiri, Brain and Mind Centre, University of Sydney, Sydney, Australia.

Manoj, P., Menezes, TY Nelson Department of Neurology and Neurosurgery, Children’s Hospital at Westmead, and Discipline of Child and Adolescent Health, University of Sydney, Sydney, Australia.

Mark, A., Paine, Department of Neurology, Royal Brisbane and Women’s Hospital, Brisbane, Australia.

Mark, Marriot, Department of Neurology, Royal Melbourne Hospital, Australia.

Marzena, J., Fabis-Pedrini, Perron Institute for Neurological and Translational Science, Centre for Neuromuscular and Neurological Disorders, The University of Western Australia, Western Australia, Australia.

Mastura, Monif, Department of Neurosciences, Central Clinical School, Monash University, Melbourne, Australia.

Michael, Boggild, Department of Neurology, Townsville Hospital, North Queensland.

Mitchell, Lawlor, Save Sight Institute, Faculty of Medicine and Health, University of Sydney, Sydney, Australia.

Monica, Badve, Department of Neurology, St George Hopsital, Sydney, Australia.

Monique, Ryan, Department of Neurology, Royal Children’s Hospital, Melbourne, Australia.

Muhammed, Aaqib, University of Western Australia, Perth, Australia.

Neil, Shuey, Neuro-ophthalmology clinic, Royal Victorian Eye and Ear Hospital and Department of Clinical Neurosciences, St Vincent’s Hospital, Melbourne, Australia.

Nerissa, Jordan, Department of Neurology, Fiona Stanley Hospital, Perth, Australia.

Nicholas, Urriola, Department of Neurology, Royal Prince Alfred Hospital, Sydney, Australia.

Nicholas, Lawn, Department of Neurology, Sir Charles Gairdner Hospital, Perth, Australia.

Owen, White, Department of Neurosciences, Monash University, Melbourne, Australia.

Pamela, McCombe, School of Medicine, University of Queensland, Brisbane, Australia.

Rakesh, Patel, Paediatric Neuroservices, Starship Children’s Health, Auckland District Health Board, Auckland, New Zealand.

Richard, Leventer, Department of Neurology, Royal Children’s Hospital, Melbourne, Australia.

Richard, Webster, TY Nelson Department of Neurology and Neurosurgery, Children’s Hospital at Westmead, Sydney, Australia.

Robert, Smith, Department of Neurology, John Hunter Children’s Hospital, Newcastle, Australia.

Sachin, Gupta, TY Nelson Department of Neurology and Neurosurgery, Children’s Hospital at Westmead, Sydney, Australia.

Shekeeb, S., Mohammad, TY Nelson Department of Neurology and Neurosurgery, Children’s Hospital at Westmead, Sydney, Australia.

Sekhar, Pillai, Department of Neurology, Sydney Children's Hospital, Sydney, Australia.

Simon, Broadley, Department of Neurology, Gold Coast University Hospital, School of Medicine, Griffith University, Gold Coast, Australia.

Simon, Hawke, Central West Neurology and Neurosurgery, Orange, and the Faculty of Medicine and Health, the University of Sydney, Sydney, Australia.

Steve, Vucic, Department of Neurology, Westmead Hospital, Sydney, Australia.

Sumu, Simon, Department of Ophthalmology, Royal Adelaide Hospital, Adelaide, Australia.

Sophie, Calvert, Department of Neurosciences, Queensland Children's Hospital, South Brisbane, Australia

Stefan, Blum, Princess Alexandra Hospital, Brisbane, Australia.

Stephen, Malone, Department of Neurosciences, Queensland Children's Hospital, South Brisbane, Australia

Suzanne, Hodgkinson, Department of Neurology, Liverpool Hospital, Sydney, Australia.

Todd, A., Hardy, Department of Neurology, Concord Repatriation General Hospital, University of Sydney, Australia.

Tomas, Kalincik, CORe, Department of Medicine, University of Melbourne, and Department of Neurology, Royal Melbourne Hospital, Melbourne, Australia.

Tyson, Ware, Department of Pediatrics, Royal Hobart Hospital, Hobart, Australia.

Victor, S. C., Fung, Department of Neurology, Westmead Hospital, Sydney, Australia.

William, Huynh, Brain and Mind Centre, University of Sydney and Prince of Wales Clinical School, University of New South Wales, Sydney, Australia.
